# Comprehensive Insights into Plant-Derived Bioactive Peptides: Sources, Technological Strategies, and Health Implications

**DOI:** 10.3390/molecules31162866

**Published:** 2026-08-17

**Authors:** Gabriela Kowalska, Gabriela Rzepkowska, Karolina Miśkiewicz, Mateusz Joachimowski, Justyna Rosicka-Kaczmarek

**Affiliations:** 1 Institute of Food Technology and Analysis, Faculty of Biotechnology and Food Sciences, Lodz University of Technology, 90-537 Lodz, Poland; 257261@edu.p.lodz.pl (G.R.); karolina.miskiewicz@p.lodz.pl (K.M.); 2 Department of General Surgery, Radom Specialist Hospital, 26-610 Radom, Poland; m.joachimowski@szpital.radom.pl

**Keywords:** bioactive peptides, plant proteins, alternative sources, enzymatic hydrolysis, functional foods, sustainability, antioxidant activity

## Abstract

Interest in sustainable protein sources is increasing because of environmental concerns related to animal agriculture and the growing burden of chronic non-communicable diseases. Plant-derived bioactive peptides (PDBAPs), amino acid sequences released from dietary proteins, are gaining attention because experimental studies have reported activities relevant to hypertension, type 2 diabetes, and cancer-associated processes. Although animal proteins have long been major sources of bioactive peptides, plant materials may offer advantages such as abundance, potentially lower production costs, and broad cultural acceptability; however, these benefits depend on the source, processing requirements, safety, and scale-up conditions. This review integrates plant sources, processing technologies, proposed mechanisms of action, and translational barriers. Current research covers traditional sources, including legumes and cereals, as well as agro-industrial by-products such as potato peels, spent coffee grounds, and broccoli stems. Modern processing strategies increasingly combine enzymatic hydrolysis or microbial fermentation with process-assisting technologies, including ultrasound treatment and subcritical water processing, to improve protein recovery or peptide release. Recent studies also examine proposed mechanisms of PDBAP activity, including Keap1/Nrf2-associated responses and inhibition of enzymes involved in metabolic disorders. Evidence is interpreted according to the stage of experimental validation, from computational prediction and cell-free assays to cellular, animal, and human studies. Key challenges remain, particularly digestive instability, uncertain systemic bioavailability, bitterness, safety standardization, and limited human clinical evidence. Future work should prioritize standardized extraction and analytical methods, optimized delivery systems, and robust clinical trials.

## 1. Introduction

Bioactive peptides (BAPs) have gained attention because of their potential to support the prevention and management of chronic diseases. Advances in analytical methods have enabled the identification of numerous peptide sequences released from dietary proteins and have revealed their diverse biochemical properties. Although animal-derived proteins have historically been the main source of BAPs, interest in plant-based systems is increasing. This transition is driven by ecological and ethical concerns, rising global protein demand, and the need for alternatives that are nutritionally adequate and culturally acceptable. An expanding global population and growing environmental pressures further underscore the need for more efficient and eco-friendly protein sources. Many plant materials, including by-products, contain proteins that can yield bioactive peptides.

Consequently, plant-derived peptides have become an important focus in the development of functional foods, nutraceuticals, and potential therapeutic agents.

### 1.1. Definition of BAPs

Bioactive peptides (BAPs) are amino acid sequences—commonly, but not exclusively, 2 to 20 residues—that exert physiological activities beyond their nutritional value [1,2]. Longer sequence-defined peptides, including lunasin, are also discussed in the food-derived bioactive peptide literature. These peptides may be present in an inactive, encrypted form within parent proteins and become active after release through enzymatic hydrolysis, microbial fermentation, chemical synthesis, or gastrointestinal digestion [3,4,5]. Low molecular mass may favor the intestinal transport of some peptides, but molecular mass alone does not demonstrate digestive stability, epithelial permeability, or the ability of an intact peptide to reach the systemic circulation [2,6]. Safety and regulatory status are specific to the source, composition, production process, dose, intended use, and jurisdiction; protein hydrolysates enriched with BAPs should therefore not be regarded as generically GRAS. The global functional protein and peptide market has been estimated at around USD 75 billion per year [7].

In this review, analytically different materials are distinguished as follows: (i) purified, sequence-confirmed native peptides; (ii) chemically synthesized peptides; (iii) molecular-weight-defined or chromatographically enriched peptide fractions; (iv) crude or minimally fractionated protein hydrolysates; and (v) intact parent proteins or source-associated components considered in relation to precursor function or safety. Activity observed for a hydrolysate or peptide fraction is not attributed to an individual sequence unless that sequence has been isolated or synthesized and independently validated. Intact allergens, lectins, protease inhibitors, ribosome-inactivating proteins, canatoxin, and other source-associated hazardous components are discussed in the safety section and are not classified as plant-derived BAPs.

Cryptic or encrypted bioactive peptides are sequences embedded within larger parent proteins that do not exert their characteristic biological activity until released by proteolysis, fermentation, gastrointestinal digestion, or controlled processing [8]. Such peptide reservoirs are not restricted to classical dietary storage proteins; enzymes and other structurally or functionally diverse proteins may also contain potentially active sequences [8]. Their release depends on the accessibility of protease-sensitive regions, the tertiary structure of the parent protein, the specificity and combination of proteolytic enzymes, and processing conditions that alter protein conformation. Computational tools such as BIOPEP-UWM can support the prediction of encrypted peptide sequences and simulate their release during proteolysis, providing a useful hypothesis-generating framework prior to analytical and biological validation. Ribosome-inactivating proteins also illustrate this broader concept, as selected plant RIPs have been reported to contain cryptic peptide sequences with antimicrobial and antibiofilm activities, demonstrating that proteins discussed primarily in a toxicological context may simultaneously serve as reservoirs of potentially beneficial bioactive motifs [9].

### 1.2. A New Perspective on the Essential Role of Plant-Based Proteins

The shift toward plant-based protein systems reflects several converging factors, including ecological responsibility, sustainability, resource efficiency, population growth, and public health recommendations. Global estimates indicate that by 2050 the world population may reach 8–10 billion people, increasing the demand for accessible, high-quality protein [5,10,11]. Animal agriculture is inefficient from a protein-conversion perspective, as only about 3% of the plant protein consumed by livestock is converted into animal protein [10,12]. Environmental considerations also contribute to this transition. Livestock production is associated with deforestation, water scarcity, and greenhouse gas emissions [10,13,14]. Producing equivalent amounts of animal protein may require up to 100 times more water and more than ten times more land than producing plant-derived protein [10,15]. As a result, plant-based diets are increasingly regarded as sustainable strategies for reducing environmental impacts [14]. Major health organizations, including WHO and WCRF, recommend reducing meat consumption in favor of plant-based dietary patterns because such diets are associated with lower risks of obesity and cardiovascular disease [10,14,16]. Beyond sustainability, plant-derived bioactive peptides (PDBAPs) offer several additional advantages. Plant-derived peptide preparations are not inherently hypoallergenic. Their allergenic potential depends on the botanical source, persistence of allergenic epitopes, processing conditions, extent of hydrolysis, purity, and susceptibility of the target population. Suitability for infants or other sensitive populations therefore requires ingredient-specific immunological, clinical, and regulatory evaluation [1,5,14]. They are also less constrained by cultural and religious dietary restrictions, making them applicable to diverse populations worldwide [1,5]. A rapidly growing area of interest involves the use of agro-industrial by-products, such as potato peels, oilseed meals, and spent coffee grounds, as potentially valuable protein sources [17,18,19]. These materials often contain valuable bioactive compounds but are commonly discarded [20]. Their valorization can reduce waste while providing promising substrates for the production of health-promoting peptides. Despite these advantages, several challenges remain.

Many peptides are rapidly degraded in the gastrointestinal tract, which may limit digestive stability, epithelial transport, or systemic exposure [5,21,22]. To address this limitation, current research increasingly uses in silico modeling and nanotechnology to support peptide discovery and investigate delivery strategies [21,22]. This review examines plant-based sources of bioactive peptides, technological strategies for their isolation, their health-promoting potential, and their current limitations.

### 1.3. Review Methodology

This article was prepared as a narrative review informed by a structured literature search. Searches were performed in Scopus, Web of Science, PubMed, ScienceDirect, and Google Scholar using combinations of the following terms: “plant-derived bioactive peptides”, “plant protein hydrolysates”, “bioactive peptides from legumes”, “bioactive peptides from cereals”, “bioactive peptides from seeds and nuts”, “agro-industrial by-products”, “enzymatic hydrolysis”, “microbial fermentation”, “ultrasound-assisted extraction”, “subcritical water hydrolysis”, “ACE-inhibitory peptides”, “DPP-IV-inhibitory peptides”, “alpha-glucosidase-inhibitory peptides”, “Keap1/Nrf2”, “antioxidant peptides”, “anticancer peptides”, “bioavailability”, “toxicity”, and “functional foods”. Boolean operators were used to combine source-, processing-, mechanism-, and application-related terms. The final database searches were performed on 30 June 2026. Google Scholar was used as a supplementary source; results were sorted by relevance, and the first 200 records for each predefined query were screened. Searches were performed using database-specific adaptations of the predefined search terms. The database-specific search strategies, searched fields, and document selection criteria are summarized in Supplementary Table S1.

Priority was given to peer-reviewed publications from 2015–2026, with older landmark studies included when they provided original mechanistic, analytical, or safety-related information. Eligible publications included original research articles, reviews, and database-based or in silico studies concerning plant proteins or plant-derived by-products as sources of bioactive peptides, methods for peptide release and identification, structure–activity relationships, health-related mechanisms, safety aspects, delivery systems, and food or pharmaceutical applications. Studies were excluded when they focused exclusively on animal-derived peptides; did not specify the protein source or peptide-generation method; reported bioactivity without a relevant biochemical, cellular, animal, or human endpoint; or provided insufficient methodological detail. Titles and abstracts were screened by the corresponding author to assess their relevance to the scope of this review. Potentially eligible publications were subsequently evaluated in full according to the predefined inclusion and exclusion criteria. When necessary, eligibility decisions and the interpretation of selected studies were discussed with the co-authors to ensure consistency of the review.

The structured literature search retrieved a large body of potentially relevant publications from the selected databases. Following the removal of obvious duplicate records, the literature-selection process involved title and abstract screening, followed by full-text evaluation of potentially eligible publications according to the predefined inclusion and exclusion criteria. Because this work was designed as a narrative review rather than a systematic review, the literature-selection strategy was intended to ensure comprehensive and critical coverage of the available evidence rather than exhaustive quantitative reporting according to PRISMA methodology.

Extracted information was synthesized qualitatively, with attention to the plant source, protein precursor, peptide sequence or molecular-weight fraction, production method, experimental model, reported biological activity, proposed mechanism of action, and translational limitations. The strength of evidence was interpreted according to the level of experimental validation by distinguishing computational predictions, in vitro assays, cell-based studies, animal experiments, and human intervention studies where available. Because the included literature was highly heterogeneous with respect to plant matrices, peptide preparations, analytical methods, and biological models, no quantitative synthesis or meta-analysis was attempted.

### 1.4. Classification of Analytical Status and Experimental Validation

The reviewed materials were classified independently according to their analytical status and stage of experimental validation. Analytical status was categorized as a crude hydrolysate, a molecular-weight-defined or chromatographically enriched fraction, a purified sequence-confirmed peptide, or a chemically synthesized peptide. Experimental validation was classified as V0, computational evidence only; V1, cell-free biochemical or physicochemical evidence; V2, cellular or ex vivo evidence; V3, animal in vivo evidence; V4, human observational or uncontrolled evidence; and V5, controlled human intervention evidence. The category denotes the most advanced experimental setting reported for the tested material and is not a formal assessment of methodological quality or risk of bias. A higher category does not compensate for inadequate controls, uncertain peptide composition, insufficient sample size, or absence of dose–response analysis.

## 2. Health-Promoting Properties

Plant-derived bioactive peptides (PDBAPs) have been reported to exhibit a wide range of biological activities in computational, cell-free, cellular, animal, and, less frequently, human studies. Their functional properties are influenced by chemical structure, including amino acid sequence, charge distribution, and hydrophobicity [1,23]. These characteristics may influence peptide interactions with enzymes, receptors, membranes, and transcriptional regulators, but the strength of mechanistic inference depends on the experimental model and the availability of direct target-engagement evidence.

The evidence described in Section 2.1, Section 2.2, Section 2.3 and Section 2.4 should not be interpreted as equally established. Molecular docking and molecular-dynamic simulations identify plausible interactions but do not demonstrate direct binding, enzyme inhibition, pathway activation, or physiological efficacy. Cell-free assays establish activity under defined conditions but do not establish gastrointestinal stability or in vivo effectiveness. Changes in protein abundance, phosphorylation, or nuclear translocation are described as pathway-associated responses unless causal involvement was confirmed by direct binding, target inhibition, knockdown, knockout, rescue, or comparable experiments.

### 2.1. Antioxidant Mechanisms

Plant-derived peptides exert antioxidant effects primarily through direct scavenging of reactive oxygen species (ROS), a mechanism strongly influenced by amino acid composition [24]. These peptides can neutralize hydroxyl radicals (OH), superoxide anions (O_2_^−^), singlet oxygen, and hydrogen peroxide, thereby limiting oxidative damage at the cellular level. Their ability to donate electrons or hydrogen atoms depends on the presence of specific amino acid residues that stabilize radical intermediates. Aromatic and hydrophobic residues, which are well represented in many plant protein hydrolysates, play particularly important roles in enhancing scavenging efficiency.

For example, soybean-derived peptides enriched in tyrosine (Tyr) or tryptophan (Trp) show notable radical-quenching capacity, a finding consistently reported in comparative studies on plant protein hydrolysates [23,25,26,27]. The structural basis of this activity relates to the electron- and hydrogen-donating properties of individual amino acids. Aromatic residues such as Tyr, phenylalanine (Phe), and Trp donate electrons to reactive radicals and stabilize the resulting intermediates through resonance structures [28,29]. Hydrophobic residues, including leucine (Leu), valine (Val), isoleucine (Ile), and proline (Pro), further support electron-transfer processes and contribute to efficient radical scavenging. Additional reducing capacity may arise from the formation of glutamate (Glu) through partial deamidation of glutamine (Gln), which introduces functional groups capable of donating electrons to neutralize reactive species [30]. The sulfhydryl group of cysteine (Cys) residues may also serve as both an electron donor and a hydrogen-atom donor, broadening the antioxidative versatility of peptide sequences [31]. Collectively, these structural features indicate that the antioxidant potential of plant-derived peptides is closely linked to amino acid composition, which governs their capacity to mitigate oxidative stress through direct radical scavenging [27]. Beyond qualitative structure–activity relationships, quantitative structure–activity relationship (QSAR) models and machine-learning approaches are increasingly being applied to predict the biological activity of plant-derived peptides. These computational methods integrate sequence-derived descriptors, physicochemical properties, amino acid composition, hydrophobicity, charge distribution, and structural features to prioritize candidate peptides before experimental validation. Although predictive performance continues to improve with the availability of larger peptide datasets, QSAR and machine-learning models remain hypothesis-generating tools and require validation using analytical, biochemical and biological experiments [32].

Another important pathway is metal ion chelation, which reduces ROS formation by restricting transition-metal-catalyzed redox cycling. Peptides containing histidine (His), cysteine (Cys), glutamate (Glu), or aspartate (Asp) can bind Fe^2+^ and Cu^2+^, thereby suppressing Fenton-type reactions that generate highly reactive hydroxyl radicals [25]. Examples include flaxseed-derived peptides, which exhibit Fe^2+^-chelating activity due to His-rich sequences, and rice bran peptides containing Cys and His residues that bind Cu^2+^ and reduce copper-mediated radical generation [33]. In chickpea protein hydrolysates, purified metal-binding peptides inhibited ROS formation by sequestering pro-oxidant metal ions, and their chelating efficiency increased with His content [34]. Similar observations were reported for cottonseed protein hydrolysates, which showed strong Fe^2+^-chelating capacity; their radical-scavenging activity was positively correlated with metal-binding ability. Amino acid analysis also indicated that these cottonseed-derived peptides are rich in glutamic acid (Glu) and aspartic acid (Asp), both of which can function as effective metal ligands [35]. Collectively, these findings highlight the importance of His, Glu, and Asp in reducing metal-catalyzed ROS generation and enhancing antioxidant potential. A further mechanism involves inhibition of lipid peroxidation. Hydrophobic peptides can associate with lipid bilayers or interfaces in emulsions, where they intercept lipid radicals and terminate propagation steps in peroxidation. Research summarizing advances in plant antioxidant applications highlights the relevance of peptide-mediated protection in lipid-rich systems [36]. In this context, corn gluten peptides have been reported to limit linoleic acid peroxidation, whereas sunflower protein hydrolysates can reduce lipid oxidation in emulsified systems—effects consistent with the hydrophobicity-driven antioxidant performance described for many plant peptides [36,37]. Plant-derived peptide preparations may also influence endogenous antioxidant defenses and cellular redox homeostasis [24]. Several studies have reported changes consistent with Keap1/Nrf2-associated signaling, including Nrf2 nuclear accumulation and increased expression or activity of antioxidant enzymes [38,39]. For the sunflower seed peptide PADVTPEEKPEV and the rice bran peptide AFDEGPWPK, computational analyses suggested possible compatibility with residues within the Keap1 Kelch domain [19,40]. These results represent predicted interaction models rather than direct evidence of peptide–Keap1 binding. Where cellular or animal studies additionally demonstrated Nrf2-associated responses, the findings support pathway association; however, confirmation of direct target engagement and causal disruption of the Keap1–Nrf2 complex requires biochemical, biophysical, or loss-of-function validation [41].

### 2.2. Antihypertensive Activity

Many plant-derived peptide preparations investigated for antihypertensive potential have been evaluated for inhibition of angiotensin-converting enzyme (ACE), a zinc-dependent dipeptidyl carboxypeptidase that plays a central role in the renin–angiotensin system (RAS) [42]. The RAS is a key hormonal cascade regulating blood pressure, fluid balance, and vascular tone. In this pathway, renin cleaves angiotensinogen to form angiotensin I (Ang I), and ACE subsequently hydrolyzes Ang I to generate angiotensin II (Ang II), a potent vasoconstrictor. ACE also degrades bradykinin, a vasodilatory peptide that promotes nitric oxide and prostacyclin release [43]. Cell-free ACE-inhibition assays demonstrate activity under the specified experimental conditions but do not independently establish blood-pressure reduction in vivo. ACE-inhibitory activity has also been reported for peptide preparations obtained from legumes, including common beans, although the majority of available evidence originates from in vitro enzymatic assays rather than clinical studies [44]. Some sequence-defined plant peptides have shown competitive ACE-inhibition kinetics, whereas the inhibition mode has not been established for many hydrolysates and peptide fractions [45]. Inhibitory activity is influenced by peptide length, charge, hydrophobicity, and the identity of residues near the C-terminus; aromatic or hydrophobic residues such as Pro, Phe, Tyr, and Trp are frequently present in active sequences, although their presence is not sufficient to predict activity. Structural studies of human ACE in complex with natural inhibitory peptides have identified the S1, S2 and S1′ subsites as key determinants of peptide recognition and binding, providing an experimental structural framework for interpreting subsequent computational docking studies [46]. Molecular-docking studies of sequences such as VTYM from ginger and YSK from rice bran have proposed possible orientations within or near the ACE catalytic region [40]. Such models indicate computational compatibility and should not be interpreted as proof of direct binding, catalytic-site occupation, or a specific inhibition mechanism. Evidence of antihypertensive action in animals or humans should be considered separately because it also depends on digestive stability, active metabolites, absorption, vascular effects, and the composition of the administered preparation. Some PDBAP-containing preparations may additionally be associated with antioxidant or endothelial responses, but these complementary mechanisms require preparation-specific validation [47]. Although this review focuses on plant-derived bioactive peptides, it is worth noting that modulation of the renin–angiotensin system can also be achieved using pharmacological agents acting through different mechanisms. Experimental formulation studies have shown that metal complexation may modify the antihypertensive properties of telmisartan, illustrating the diversity of therapeutic strategies targeting the RAS [48].

### 2.3. Antidiabetic Potential

Plant-derived peptide preparations have been investigated against several targets relevant to postprandial glucose regulation. In the intestine, inhibition of alpha-amylase and alpha-glucosidase may slow carbohydrate hydrolysis and glucose release, whereas DPP-IV inhibition may prolong incretin activity [49,50,51]. These cell-free assays identify inhibitory activity under defined conditions, but results depend strongly on the enzyme source, substrate, assay format, concentration range, and comparator and should not be compared directly across studies. Inhibition of luminal carbohydrate-digesting enzymes represents a potential local mechanism that does not necessarily require systemic absorption. By contrast, proposed effects on insulin-receptor signaling, GLUT4 translocation, peripheral insulin sensitivity, or GLP-1 secretion require stronger cellular, in vivo, and exposure-related evidence [52,53,54,55]. Peptides from hemp-derived preparations and fermented spent coffee grounds have been examined using combinations of enzyme assays and computational modeling [16]. For the spent-coffee-ground-derived sequences YGF and GMCC, high percentage inhibition was reported at approximately 0.42 mg/mL; however, interpretation requires the analytical form and purity of the tested material, enzyme source, assay conditions, positive control, dose–response relationship, and IC50 value where available [16]. Docking analyses suggested possible orientations near alpha-glucosidase or alpha-amylase catalytic regions, but these predictions do not establish direct binding or inhibition mechanism. Cellular changes involving the IR/IRS-1/AKT pathway, GLUT4, or GLP-1 are therefore described as pathway-associated observations unless causal target dependence was demonstrated. Overall, these findings identify candidates for further nutraceutical research but do not yet establish clinical efficacy in type 2 diabetes.

### 2.4. Immunomodulatory and Anticancer Effects

Plant-derived peptide sequences have been investigated for immunomodulatory activity, and short motifs such as YG, YGG, GLF, TPRK, GVM, GFL, EAE, and LLY are catalogued in the BIOPEP-UWM database [56]. Database annotation and in silico proteolysis are useful for hypothesis generation but do not establish peptide release in a food matrix or immunological activity. Food-grade proteases such as bromelain and ficin can release low-molecular-weight fractions from cereal and legume proteins, including rice proteins and bran [57]. In a cellular study, the rice-derived peptide GBP1 (NSVFRALPVDVVANAYR) was associated with dendritic-cell maturation, changes in CD86 and MHC II expression, altered cytokine production, and increased IL-4/IL-10 levels [54]. Molecular docking additionally suggested possible compatibility with an MHC-II binding region; this result is a computational prediction and not evidence of direct MHC-II binding. Confirmation of an MHC-II-dependent mechanism would require direct binding measurements and functional blocking or loss-of-function experiments. Collectively, database mining, protease-guided release, and selected cell-level observations support further investigation of plant-derived immunomodulatory peptides, but the evidence remains preparation- and model-specific [56,57,58].

A prominent example is lunasin, a 43-residue peptide first isolated from soybean and later detected in wheat and barley. Lunasin contains an RGD motif and an acidic C-terminal domain and has been investigated in relation to histone acetylation, cellular transformation, and experimental carcinogenesis [59,60,61]. These activities have been evaluated in biochemical, cellular, and selected animal models, but the contribution of intact orally administered lunasin, its digestive products, and the food matrix remains an important translational question [62]. Rice-bran peptide preparations and the purified pentapeptide EQRPR have reduced viability or proliferation in several cancer-cell-line assays [63]. Percentage inhibition values should be interpreted together with peptide purity, concentration, exposure duration, assay format, comparator, replication, and IC50 values where reported. Similarly, the walnut-derived peptide CTLEW was associated with reduced viability and markers of apoptosis or autophagy in selected cell models [64]. Lower toxicity toward one non-tumor cell line under the tested conditions may indicate differential cellular sensitivity but is insufficient to establish general tumor selectivity or clinical safety. These studies identify candidates for further mechanistic and in vivo investigation; they do not demonstrate cancer-preventive or therapeutic efficacy in humans [59,60,63,64].

The main molecular targets, structural determinants, and reported stages of experimental validation for the major health-promoting effects of plant-derived bioactive peptides are summarized in Table 1. Study-level analytical and experimental details are provided in Supplementary Table S2.

## 3. Alternative Plant-Based Sources of Bioactive Peptides

Plant-based protein sources represent a broad and still underutilized reservoir of bioactive peptides (BAPs). They include widely cultivated crops as well as agricultural by-products that are often overlooked or discarded. Each botanical group contains proteins with distinct structural characteristics, enabling the release of peptides with diverse biological functions. Plant-derived proteins therefore provide an important foundation for the development of functional foods and nutraceutical formulations [22,26]. The examples discussed below include sequence-confirmed peptides, molecular-weight-defined fractions, and crude hydrolysates. These analytical categories are identified separately, and activity observed for a complex preparation is attributed to that preparation rather than automatically to an individual detected sequence.

### 3.1. Legumes

Legumes constitute one of the most significant non-animal protein sources, with typical protein concentrations ranging from 20% to 40% [23]. Soybean is within this group. It restores endogenous insulin-producing mass by promoting pancreatic beta-cell proliferation while inhibiting apoptosis and dedifferentiation under glucolipotoxic conditions. Long-term Vglycin treatment also improves insulin sensitivity, reduces systemic inflammatory markers (IL-1beta, IL-6), and enhances metabolic homeostasis [68]. Other legumes, such as chickpeas (up to 32.6% protein) and peas (up to 30.9% protein), are also important reservoirs of bioactive sequences. Chickpea-derived peptides exhibit antiproliferative activity by inhibiting growth of endometrial and breast cancer cells through cell-cycle arrest, caspase-dependent apoptosis, and p53 upregulation [69,70]. Similarly, pea-derived peptides activate the Keap1/Nrf2 pathway to support cellular antioxidant defenses and release specific sequences such as ADLPGLK during gastrointestinal digestion [23,71]. Lupin-derived peptides have also been found to inhibit HMG-CoA reductase and enhance LDL uptake, suggesting a natural pathway for lipid regulation [72]. *Glycine max* remains the most intensively characterized matrix, with certain genotypes containing up to 44.4% protein [73]. This high protein density allows for the release of specialized sequences such as lunasin, a 43-amino-acid peptide encoded within the 2S albumin gene (GM2S-1). Lunasin is recognized for its stability and bioavailability and can modulate epigenetic regulation by inhibiting histone acetylation. This mechanism contributes to suppression of oncogenic cell transformation, alongside its antioxidant and anti-inflammatory activities [60,66,74]. Beyond epigenetic modulation, soy-derived peptides such as Vglycin show metabolic regulatory effects. Vglycin has been shown to normalize fasting glucose levels in type 1 and type 2 diabetic animal models by activating insulin.

### 3.2. Cereals and Pseudocereals

Cereals and pseudocereals are abundant, economically accessible, and cost-effective matrices for bioactive peptide extraction. Although these sources generally contain less protein than legumes, typically 6.0–23%, their industrial by-products are highly valuable for peptide isolation. Rice seeds, for instance, contain 5.8–8.4% protein, whereas rice bran, a milling by-product, can be used as a source of the tripeptide YSK [75]. This sequence acts as a potent ACE inhibitor by interacting strongly with the enzyme’s active pockets [40]. Rice bran peptides have also demonstrated 80–84% growth inhibition in human colon and breast cancer cell lines [63].

Similarly, maize typically contains 9–11% protein. Corn gluten meal and zein are protein-rich maize fractions that have been used to generate peptide-containing hydrolysates; specific sequence-level effects should be reported only when supported by the corresponding primary study. Corn peptide preparations have also attenuated ethanol-induced liver injury in experimental models, with reduced hepatocyte apoptosis proposed as one contributing mechanism [76]. Other major cereals, such as wheat and oats, provide additional peptide-containing preparations; wheat germ yields peptides such as KELPPSDADW, which shows cytotoxic activity against A549 lung cancer cells [77], whereas oat-derived peptides have been clinically associated with reduced circulating IL-6 levels and decreased muscle soreness after intense eccentric exercise [78]. Pseudocereals, including quinoa (11–19% protein) and amaranth (13.1–21.5% protein), have attracted attention because of their naturally gluten-free profiles [79]. During digestion, these matrices facilitate the release of DPP-IV and alpha-glucosidase inhibitors, which are relevant to glycemic regulation in type 2 diabetes [61]. Pseudocereal-derived peptides also show in vitro chemopreventive properties [80,81].

### 3.3. Seeds and Nuts

Oilseeds and nuts can also be valuable protein sources, with protein contents ranging from 2.27 to 51.85 g per 100 g in coconut kernel and milk thistle defatted flour and from 7.50 to 21.56 g per 100 g in pecan and Virginia peanut, respectively [82,83]. Flaxseed meal contains peptides such as PFFWLHHT, which inhibit myeloperoxidase (MPO) by occupying its catalytic cavity and reducing inflammatory oxidant production [84]. Sunflower seed peptides have been shown to activate Keap1/Nrf2 signaling and enhance endogenous antioxidant responses [19]. Perilla seed meal, often discarded after oil extraction, contains hydrophobic peptide sequences such as ISPRILSYNLR, which exhibit strong lipid radical-scavenging activity [23]. Proteins have been investigated as potential precursors of multifunctional peptide preparations, but exact sequence-level claims require direct support from the corresponding primary study. Chia seeds are another promising source, containing proteins that release peptides capable of modulating the renin–angiotensin system and inhibiting key carbohydrate-digesting enzymes [85,86]. Walnut peptides have shown neuroprotective properties, including enhanced autophagy in neuroblastoma cells and improved memory performance in cognitive impairment models [87,88]. Moringa oleifera leaves and seeds provide multifunctional peptides with immunomodulatory, antimicrobial, and bone-supportive properties. Among these, DPYLGK promotes osteoblast differentiation and helps reduce bone resorption [27].

### 3.4. Undervalued Plants and Industrial Waste as Alternative Sources of Protein

Upcycling agro-industrial by-products may contribute to circular-bioeconomy strategies, as these waste materials may contain higher concentrations of bioactive compounds than the primary edible parts of the plant. For instance, while potato tubers contain approximately 3% protein, potato peels, which represent about 40% of processing waste, contain 8.0–17.1% protein on a dry weight basis [89]. These peels are rich in patatin, which can be hydrolyzed into anti-inflammatory and cardioprotective peptides. Potato protein-derived peptides may also be generated through endogenous proteolytic activity in different parts of the potato tuber, producing preparations with measurable ACE-inhibitory activity [90]. Similarly, the approximately 6 million tons of spent coffee grounds (SCG) generated annually have been investigated as fermentation substrates to yield peptides such as YGF and GMCC, which demonstrate more than 89% inhibition of alpha-glucosidase activity [91]. The use of agricultural and food-processing by-products may expand the range of substrates available for peptide discovery and valorization. Potato peels are rich not only in patatin but also in protease inhibitors that can release peptides with anti-inflammatory and cardioprotective properties, in preclinical models [17]. Broccoli stems, typically regarded as low-value residues, contain proteins capable of generating bioactive peptides such as KSVLLKF, which stimulates keratinocyte proliferation and supports wound healing [92]. Fermented spent coffee grounds provide peptides such as YGF and GMCC, which exhibit more than 89% inhibition of alpha-glucosidase activity [18], consistent with earlier findings on SCG-derived peptides [91]. Additional underutilized biomass includes tomato processing waste, which yields antioxidant peptides capable of protecting DNA from hydroxyl radical-induced damage [29], and marine algal species such as Gracilariopsis lemaneiformis and Ulva sp., which provide ACE-inhibitory and radical-scavenging peptides. Collectively, these undervalued plant materials and industrial by-products may provide potentially valuable substrates, subject to verification of recovery yield, safety, environmental performance, downstream purification requirements, and economic feasibility [26]. Net environmental performance depends on protein-recovery yield, enzyme and energy demand, water consumption, pressure or temperature requirements, stabilization, transport, purification, wastewater generation, and contaminant control. Life-cycle assessment, techno-economic analysis, or pilot-scale comparison is therefore required before a process can be described as green, low-cost, or industrially sustainable.

Representative plant sources and agro-industrial by-products, together with analytically defined peptide preparations, production methods, reported activities, experimental models, stages of validation, and key limitations, are summarized in Table 2. Each sequence-defined peptide or clearly defined fraction is presented separately to avoid implying associations between unrelated sources, methods, and outcomes.

## 4. Production, Fractionation, Identification, and Validation of Peptides

The release of bioactive peptides (BAPs) from parent proteins is essential for their biological activity because these sequences remain inactive within intact protein structures. The release method strongly influences molecular size, sequence composition, and functional properties [1,2]. Although enzymatic hydrolysis and microbial fermentation have traditionally dominated peptide production, the integration of emerging green technologies has increased both process efficiency and peptide yield [98,99]. In practice, peptide development is a multistep workflow that may include raw-material stabilization, protein extraction, peptide release, fractionation, purification, LC-MS/MS identification, database searching or de novo sequencing, synthetic confirmation, purity assessment, and biological revalidation. Technologies acting at different stages should not be presented as functionally equivalent.

### 4.1. Protein Recovery and Pretreatment

Peptide production commonly begins with recovery or enrichment of the parent protein. Operations may include milling, stabilization, defatting, aqueous or alkaline extraction, isoelectric precipitation, membrane concentration, and drying. These conditions influence protein yield, denaturation, solubility, co-extraction of phenolic compounds or contaminants, and subsequent susceptibility to proteolysis. Ultrasound and high hydrostatic pressure are generally considered pretreatment or process-intensification technologies because they may disrupt plant structures or alter protein conformation without necessarily cleaving peptide bonds.

### 4.2. Enzymatic Hydrolysis

Enzymatic hydrolysis is the most widely used method for producing BAPs in laboratory and industrially relevant processes because of its specificity, mild processing conditions, and avoidance of strong chemical hydrolysis reagents [2,4]. Proteases selectively cleave peptide bonds, reduce allergenicity, and improve the nutritional properties of the resulting hydrolysates [2]. Enzyme selection is critical because different proteases recognize distinct cleavage sites. Broad-specificity serine endopeptidases such as alcalase are frequently applied to plant proteins, including wheat, corn, and soy, owing to their high catalytic efficiency [100]. Plant-derived cysteine proteases such as papain and bromelain are also widely used because they release small peptides with notable antioxidant and antihypertensive activities [2,101,102]. To diversify peptide profiles, many studies apply sequential hydrolysis, in which proteins are treated with two or more enzymes simultaneously or sequentially. This approach often produces smaller peptide fragments with enhanced multifunctional properties [85,103]. The resulting peptide profile depends on the parent protein, enzyme specificity, enzyme-to-substrate ratio, pH, temperature, reaction time, degree of hydrolysis, and enzyme-inactivation method. Enzyme treatment alone does not establish the presence or activity of a specific peptide; sequence identification and independent validation are required for sequence-level attribution.

### 4.3. Microbial Fermentation

Microbial fermentation provides a potentially economical route for peptide generation by using species- and strain-dependent proteolytic systems [4]. These systems may include extracellular enzymes, cell-envelope-associated proteinases and peptidases, transport components, and intracellular peptidases; their organization and specificity differ among bacteria and fungi. Fermentation may simultaneously alter antinutritional factors, allergenicity, acidity, flavor, and microbial safety [104]. Lactic acid bacteria, including Lactiplantibacillus plantarum and Levilactobacillus brevis, are commonly investigated for fermenting legumes and cereals [92,93]. Fermentation may reduce bitter off-notes in some preparations, but effects depend on the strain, substrate, fermentation conditions, and subsequent processing. Mixed-culture fermentation may yield a broader peptide profile than a single-strain system because of complementary proteolytic activities [105].

### 4.4. Process-Assisting Technologies

To improve protein extraction, enzyme accessibility, or hydrolysis efficiency, several physical technologies have been incorporated into peptide-production workflows [99]. These treatments assist particular process stages and should not be interpreted as equivalent to enzymatic or microbial peptide-bond cleavage.

Ultrasound-Assisted Extraction (UAE)

UAE applies acoustic cavitation forces that disrupt cell walls and alter protein tertiary structure, which may improve protein extraction or expose cleavage sites for subsequent enzymatic hydrolysis [7,106].

This process uses high-frequency sound waves (>16 kHz) that are undetectable by the human ear. UAE enhances enzyme–substrate interactions and may reduce extraction or hydrolysis time under optimized conditions. It has been used to increase antioxidant peptide yields from flaxseed and soybean meal [35,84].

High Hydrostatic Pressure (HHP)

HHP subjects samples to pressures of 100–1000 MPa, promoting protein unfolding and structural relaxation [107]. This may facilitate subsequent enzymatic release of peptides from structurally resilient proteins, such as those in potatoes and sweet potatoes, and often enhances ACE-inhibitory and antioxidant activities [29].

Subcritical Water Processing (SWP)

SWP uses water under high temperature (100–374 °C) and pressure (10–220 bar), conditions that increase water’s ionic product and promote protein extraction, hydrolysis, or both, depending on the processing conditions [108]. SWH has proven particularly effective for processing oilseed by-products such as chia expeller, producing peptide-rich extracts with improved bifunctional properties compared with alkaline extraction methods [86]. Excessive temperature or residence time may also promote amino-acid degradation, racemization, oxidation, or Maillard-type reactions; therefore, SWP should not be described as inherently mild or universally green without process-specific yield, energy, and product-quality data. At industrial scale, process optimization also depends on engineering factors such as efficient heat transfer, temperature control, and energy management. These technological aspects influence process performance but should be considered separately from the biological activity of the resulting peptide preparations [109].

### 4.5. Fractionation, Identification, and Biological Confirmation

Crude hydrolysates commonly contain residual proteins, peptides of different sizes, free amino acids, salts, enzymes, phenolic compounds, carbohydrates, and source-associated contaminants. Ultrafiltration can separate broad molecular-weight fractions but does not provide peptide-level purity. Further purification may involve size-exclusion, ion-exchange, affinity, or reversed-phase chromatography. Peptide-rich fractions are commonly analyzed by LC-MS/MS using database searching, de novo sequencing, or both. Where feasible, sequence assignment should be confirmed using authentic synthetic peptides before biological activity is attributed to an individual peptide. Detection of a sequence does not demonstrate that it is responsible for the activity of a fraction. Definitive attribution of biological activity to an individual peptide requires purification or chemical synthesis, confirmation of identity and purity, dose–response testing, appropriate controls, and biological revalidation in the relevant model. The principal stages used to recover, release, fractionate, identify, and validate plant-derived bioactive peptides are compared in Table 3.

## 5. Challenges and Limitations

Despite the substantial potential of BAPs, several barriers continue to limit their broader industrial application and clinical translation. These challenges include sensory drawbacks, low metabolic stability, safety considerations related to plant constituents and by-products, and technological and regulatory constraints [1,17]. Understanding these limitations is crucial for optimizing peptide production and facilitating commercialization.

### 5.1. Sensory Attributes and Bitterness

A major obstacle to incorporating BAPs into food products is the bitterness that often develops during enzymatic hydrolysis [1,112]. Peptide hydrophobicity and amino-acid composition influence bitter taste, and structural characteristics associated with ACE inhibition may partially overlap with those associated with bitterness [113]. Hydrolysis exposes hydrophobic amino acids such as Phe, Leu, Ile, and Tyr, which are frequently associated with bitter taste [1]. Bitterness tends to increase with a higher degree of hydrolysis and with hydrophobic residues at the C-terminus or bulky basic residues at the N-terminus. For example, peptides from flaxseed protein hydrolysates smaller than 1 kDa were found to be more bitter than larger fragments. Strategies to address this issue include the use of sweeteners, encapsulation techniques, and targeted exopeptidase treatments designed to remove or modify bitter residues [112]. Recent research has highlighted microbial fermentation as a potential bioprocessing strategy for plant-based matrices. Studies on pea protein-based beverages demonstrate that precise selection of microbial cultures can improve sensory profiles by reducing bitter off-notes and enhancing flavor volatiles. Nevertheless, optimized and standardized fermentation protocols remain critical for industrial scalability and product consistency [110].

### 5.2. Digestive Stability, Bioaccessibility, Intestinal Transport, and Systemic Bioavailability

Several distinct processes determine whether an orally consumed peptide can reach its proposed biological target. Digestive stability refers to resistance to transformation or degradation during gastric and intestinal digestion. Bioaccessibility refers to the fraction released from the food or delivery matrix and available for interaction with the intestinal environment. Epithelial permeability or transepithelial transport describes passage across the intestinal epithelium or an epithelial model. Systemic bioavailability refers to the fraction reaching the systemic circulation in an intact or biologically relevant form, whereas pharmacokinetics describes concentration over time, distribution, metabolism, and elimination. Many BAPs are susceptible to degradation by pepsin, trypsin, chymotrypsin, brush-border enzymes, or intracellular peptidases [21]. Low molecular mass may favor transport of some peptides but does not itself demonstrate absorption. The general statement that systemic bioavailability is often below 10% has therefore been removed because quantitative exposure is peptide- and method-specific. The standardized INFOGEST protocol reproduces oral, gastric, and intestinal digestion phases and is useful for evaluating release, transformation, and digestive stability [114,115,116]. It does not demonstrate epithelial transport or systemic exposure. These processes require complementary experimental approaches, including validated intestinal-cell models, ex vivo tissue systems, portal or systemic blood measurements, isotope-labelled peptides, and targeted mass-spectrometric pharmacokinetic analyses. Biological target location is also important: peptides proposed to inhibit alpha-amylase or alpha-glucosidase within the intestinal lumen may act locally without systemic absorption, whereas proposed vascular, peripheral metabolic, neural, bone, or tumor effects generally require systemic exposure or generation of active metabolites. Some active sequences may be degraded during digestion, whereas others are generated only during digestion, as reported for quinoa- and pea-derived preparations [1,117]. Protective formulations, including selected liposomal or polymeric carriers, may improve digestive stability for specific preparations, but enhanced intestinal uptake or systemic bioavailability must be demonstrated directly rather than inferred from in vitro release alone [33,66].

### 5.3. Safety and Toxicological Concerns

Safety assessment of plant-derived peptide preparations must be source-, process-, composition-, dose-, use-, and population-specific. Protein hydrolysates and peptide preparations are not generically safe or GRAS as a class. Relevant hazards should be distinguished as IgE-mediated food allergy, HLA-DQ2/8-associated celiac immunogenicity, naturally occurring plant toxins and antinutritional proteins, processing-induced compounds, and contaminants co-extracted from by-products. Hydrolysis may reduce some allergenic determinants, but residual or newly exposed epitopes may remain. Processing may also generate compounds such as biogenic amines, lysinoalanine, D-amino acids, oxidation products, or Maillard-reaction products. In silico toxicity classifiers may support preliminary screening of sequence-defined peptides, but amino-acid-frequency patterns derived from such tools should not be presented as universal biological rules of peptide safety [118].

Growing reliance on agro-industrial by-products introduces additional risks. Potato peels contain glycoalkaloids, including alpha-solanine and alpha-chaconine, which can cause gastrointestinal symptoms when exposure exceeds approximately 1 mg/kg body weight [17,96]. Legume proteins may retain allergenic motifs even after hydrolysis [23].

Additional concerns include pesticide residues, mycotoxins, heavy metals, microbial contamination, packaging-derived substances, process residues, and, depending on the processing history, polycyclic aromatic hydrocarbons (PAHs) [119]. These hazards require raw-material specifications, validated decontamination procedures, traceability, and batch testing.

Several plant sources contain well-defined allergenic proteins, immunogenic peptides, or toxic proteins that must be distinguished from the target BAP preparations. Gluten-containing cereals provide the hydrolysis-resistant alpha2-gliadin 33-mer, which is central to celiac disease [120,121,122,123]. Major allergens, such as Gly m 4, Gly m 5, and Gly m 6 in soybean and Ara h 1, Ara h 2, Ara h 3, and Ara h 6 in peanut, can elicit severe IgE-mediated reactions [31,118,124,125]. Enzymatic hydrolysis of roasted peanut protein extract may reduce IgE-binding activity, although the extent of reduction depends on the enzyme and hydrolysis conditions, and residual immunoreactivity may remain [126]. Additional concerns include canatoxin from jack bean [127,128,129], proteins with predicted allergenic potential [130], and ribosome-inactivating proteins (RIPs) and lectins that are widespread in plants [131,132,133,134,135]. Highly toxic stenodactylin further illustrates the need for careful monitoring [136].

Proteolytic processing may reduce the abundance of some allergenic epitopes or lectin activity, but the effect is source-, enzyme-, and process-dependent [87,137,138,139,140,141]. IgE-mediated food allergy and celiac disease should be considered separately: the former involves allergen-specific IgE and may be triggered by linear or conformational epitopes of different sizes, whereas the latter involves HLA-DQ2- or HLA-DQ8-restricted T-cell responses to specific gluten-derived sequences [120,121,122,123,124,125]. No universal minimum peptide length defines IgE reactivity. Sequence-screening resources such as AllergenOnline and celiac-peptide databases can support hazard identification but cannot replace immunological or clinical evaluation [142].

Safety strategies include selecting low-allergenic sources; applying hydrolysis, heat treatment, or fermentation to reduce allergenicity [143,144,145]; and using fractionation or purification to remove hazardous components. The development of hypoallergenic cultivars and source-specific processing strategies may reduce risk for selected ingredients [145]. Regulatory oversight and accurate allergen labeling are critical for ensuring the safe use of PBPs in foods [146]. Regulatory evaluation should consider the botanical source, manufacturing process, production organisms and enzymes, compositional specifications, residual proteins, contaminants, intended dose, target population, allergen labelling, and applicable jurisdiction.

The principal translational barriers, possible mitigation strategies, and remaining evidence gaps are summarized in Table 4.

## 6. Current and Prospective Applications in Food, Nutraceutical, and Biomedical Research

Bioactive peptides derived from plant proteins are increasingly explored for potential roles across the food, nutraceutical, and pharmaceutical sectors. The maturity of evidence differs substantially among these applications. Incorporation into a food matrix demonstrates technical feasibility but does not itself establish a human health benefit, and activity in an enzyme assay, cell line, or animal model does not establish clinical efficacy [10].

### 6.1. Functional Foods and Nutraceuticals

One of the most immediate and commercially relevant uses of plant-derived BAPs is the formulation of functional foods. Beyond reported bioactivities, peptides exhibit a range of techno-functional properties that can be leveraged in food systems. These molecules may serve as versatile hydrocolloids and additives, functioning as high-intensity sweeteners, pigment stabilizers, and rheological modifiers [10]. Their surface-active nature also allows them to act as emulsifiers and encapsulants, while their chemical structure contributes to flavor enhancement, acidity control, and anti-caking effects by preventing crystalline or powder aggregation [143]. For example, chia-derived antioxidant peptides have been incorporated into bread formulations. Adding up to 10 mg of peptides per gram of flour enhanced bread texture and maintained high antioxidant activity after baking and extended storage [150]. Plant-derived peptide preparations have also been investigated in beverages. Pistachio-based drinks and fermented soy beverages have been enriched with peptide fractions to improve stability and retain assay-based activities such as ACE inhibition or glucose-regulation-related endpoints [152]. Beyond fortification, BAPs can contribute to food preservation. Corn protein hydrolysates, for example, inhibit lipid oxidation in ground meat products, extending shelf life without synthetic additives [153]. Their ability to delay oxidative degradation supports their investigation as natural preservative ingredients in clean-label food formulations. Strategies to enhance peptide functionality and stability and biological performance after ingestion include absorption enhancers, structural modifications, protease inhibitors, and colloidal delivery systems such as liposomes, emulsions, and nanoparticles. Recent advances also highlight targeted enzymatic hydrolysis and fermentation as effective methods for generating bioactive peptides with improved functional properties [154].

### 6.2. Preclinical and Clinical Relevance

Advances in peptidomics are expanding understanding of plant-derived peptide candidates, but most evidence remains at the discovery or preclinical stage. Cell-free enzyme inhibition, antioxidant assays, molecular docking, and cell-culture studies are useful for candidate selection but provide limited information regarding oral efficacy, systemic exposure, effective dose, long-term safety, or clinical benefit [143,151]. Reports of anxiolytic-, antidepressant-, antihypertensive-, antimicrobial-, or anticancer-related effects should therefore be interpreted according to the tested material and experimental model [42,45,66,67,155,156]. Effects observed after administration of a whole protein isolate, complex hydrolysate, fermented product, or peptide-enriched food are attributed to the administered preparation and not to an individual sequence unless that sequence was characterized, quantified, and independently evaluated. Cytotoxicity or reduced proliferation in a cancer-cell line does not establish antitumor selectivity, clinical safety, or therapeutic efficacy. Similarly, outcomes reported with oat protein or other complex interventions cannot automatically be attributed to a specific peptide. Available controlled human evidence is limited and is summarized in Table 5, including the intervention material, participant number, dose, duration, comparator, outcome, and major limitations. Where no controlled human evidence was identified for a proposed effect, this absence is stated explicitly.

### 6.3. Delivery Strategies for Plant-Derived Bioactive Peptide Preparations

Delivery approaches should be distinguished according to the role of the peptide and the carrier. A plant-derived bioactive peptide may be the active compound being delivered; in contrast, a cell-penetrating peptide may function as a transport vector, a self-assembling peptide may form a hydrogel or carrier material, and liposomes or chitosan nanoparticles are non-peptide delivery systems. Evidence from generic peptide-delivery platforms cannot be treated as direct evidence that a dietary plant-derived BAP has been successfully delivered. Direct evidence requires incorporation of a characterized plant-derived peptide or peptide preparation into a defined carrier, followed by assessment of loading efficiency, stability, release, retained biological activity, and preferably gastrointestinal, epithelial-transport, or in vivo performance. Selected nanoliposomal systems have been experimentally evaluated with flaxseed-derived peptide fractions [33], but the direct evidence base for sequence-defined plant-derived BAPs remains limited. Generic cell-penetrating peptides and peptide hydrogels are therefore discussed only as prospective platforms unless the plant-derived BAP was actually used as the delivered active ingredient. Improved particle stability or in vitro release does not demonstrate enhanced systemic bioavailability or clinical efficacy; this requires transport or pharmacokinetic measurements.

## 7. Future Research Directions

The field of plant-derived bioactive peptides is at a pivotal stage, with increasing interest in moving from laboratory-based discovery toward reproducible and potentially scalable applications [22,151]. However, isolation, quality control, standardization, and the feasibility of using these peptides for health-related purposes remain challenging. One critical need is validation of bioactivity through in vivo studies and human clinical trials, as most current evidence is derived from in vitro models [17,67]. Although many reported peptide sequences or preparations exhibit antioxidant or inhibitory activities under controlled laboratory conditions, their biological impact in humans is often reduced by low bioavailability and rapid metabolic clearance [21]. Future studies should therefore establish effective doses, long-term safety profiles, optimal dosage forms, and pharmacokinetic behavior in human subjects [148]. Standardization represents another major research priority. Variability in extraction protocols, simulated digestion models, and analytical approaches leads to inconsistent peptide yields and bioactivity results across studies [116,151]. Streamlining these methodologies will be essential for regulatory approval and ingredient-specific regulatory assessment and potential commercialization of PDBAP preparations [150]. This remains challenging because plant matrices are highly diverse and peptide molecular diversity is incompletely characterized. Another promising direction is the development of selenium-enriched plant proteins and peptide preparations as specialized functional ingredients. Their biological properties depend on selenium speciation, protein source, processing conditions, and bioavailability, highlighting the need for comprehensive analytical characterization before potential nutritional or therapeutic applications [82]. Advances in artificial intelligence and machine learning provide valuable tools for accelerating peptide discovery. These technologies support the rational design of peptide sequences with improved stability, potency, and sensory properties, reducing reliance on trial-and-error laboratory screening [11,21,22]. Integrating computational prediction with high-throughput experimental validation may streamline development pipelines. Upcycling agro-industrial by-products also may offer opportunities for peptide sourcing within circular-bioeconomy frameworks. Future studies should also report peptide recovery yield, production efficiency and process economics using standardized metrics to facilitate comparison among production systems and support industrial translation. Waste streams such as potato peels, oilseed meals, and spent coffee grounds contain proteins that may be recovered as functional ingredients, subject to yield, safety, environmental, and economic evaluation [17,18]. Future research should focus on integrated biorefinery approaches that recover not only proteins and peptides but also other high-value compounds, such as phenolics and dietary fibers [19,86]. Finally, improved delivery systems will be crucial for enhancing PDBAP stability and bioavailability. Technologies such as nanoliposomes, chitosan nanoparticles, and pH-sensitive hydrogels show promise for shielding peptides from digestive enzymes and enabling targeted release in specific regions of the gastrointestinal tract [5,11,33]. Continued development of these systems should be evaluated as one component of translation, together with target-specific exposure and efficacy studies.

## 8. Limitations of the Present Review

This review has several limitations. Although the literature search was structured and documented, the article was designed as a narrative rather than a systematic review, and a formal study-level risk-of-bias assessment was not conducted. Database selection, search terminology, language restrictions, and incomplete indexing may have resulted in relevant publications being missed. The available studies were highly heterogeneous with respect to botanical source, protein-extraction conditions, hydrolysis method, degree of purification, peptide-identification confidence, assay design, concentration or dose, comparator, and biological endpoint. This heterogeneity precluded quantitative synthesis and direct comparison of effect sizes. Both primary studies and review articles were considered, although primary publications were prioritized for exact sequences, quantitative results, and mechanistic conclusions. Some original studies incompletely reported peptide purity, dose–response relationships, assay replication, or pharmacokinetic information. Finally, the V0–V5 classification used in this review describes the stage of experimental validation and is not a formal measure of study quality; controlled human interventions and peptide-specific pharmacokinetic data remain scarce.

## 9. Conclusions

Alternative plant-based sources, ranging from commonly consumed legumes and grains to underutilized industrial by-products, represent a broad reservoir of proteins capable of generating peptide-containing preparations. Reported antioxidant, antihypertensive, antidiabetic, immunomodulatory, and antiproliferative effects are associated with different analytical materials and stages of validation. Purified sequence-confirmed peptides, synthetic peptides, molecular-weight fractions, and crude hydrolysates should not be interpreted as equivalent, and computational prediction, cell-free assays, cellular responses, animal studies, and human interventions support different levels of inference. Process-assisting technologies such as ultrasound and high hydrostatic pressure may improve extraction or enzyme accessibility, whereas enzymatic hydrolysis, fermentation, and condition-dependent subcritical-water processing may release peptides. Environmental sustainability and industrial feasibility require process-specific evidence rather than inference from the use of plant material or by-products alone.

Despite these developments, several challenges remain. Sensory limitations such as bitterness, together with digestive instability, uncertain epithelial transport, limited peptide-specific pharmacokinetic evidence, allergenicity, contaminants, and process variability, restrict practical application. Bioinformatic tools can accelerate candidate selection, and delivery technologies may protect selected preparations, but computational prediction and formulation performance must be followed by sequence confirmation, direct biological validation, transport assessment, and in vivo or human studies. This review also has several limitations. The available literature is highly heterogeneous with respect to plant sources, protein substrates, peptide preparation methods, analytical characterization, biological models, and outcome measures. Many studies investigate complex hydrolysates or peptide-rich fractions rather than sequence-confirmed peptides, and direct comparisons among studies are often limited by differences in experimental design, assay conditions, and reporting standards. Consequently, the conclusions presented here should be interpreted according to the level of available evidence and should not be regarded as equally applicable to all plant-derived peptide preparations.

Overall, plant-derived bioactive peptides and peptide-containing preparations remain promising candidates for future functional-food and nutraceutical research. Most reported activities are supported primarily by computational, biochemical, cellular, or animal evidence, whereas direct target-engagement studies, peptide-specific pharmacokinetics, and controlled human interventions are limited. Progress will require integrated workflows combining controlled protein recovery, peptide generation, fractionation, sequence and purity confirmation, synthetic revalidation, physiologically relevant models, ingredient-specific safety assessment, and well-designed controlled human intervention studies. Such an approach will help distinguish exploratory bioactivity from reproducible and translationally relevant effects.

## Figures and Tables

**Table 1:** Biological activities, mechanisms of action and validation levels of representative plant-derived bioactive peptides.

Biological Effect	Main Molecular Targets/Pathways	Structural Features Linked with Activity	Representative Sources or Peptides	Representative Validation Approaches	Highest Level of Evidence	Representative References
Antioxidant and redox-protective activity	Direct ROS scavenging; Fe^2+^/Cu^2+^ chelation; inhibition of lipid peroxidation; potential modulation of the Keap1/Nrf2 pathway	Aromatic residues (Tyr, Trp, Phe); His, Cys, Glu and Asp involved in metal chelation; hydrophobic residues associated with lipid-phase protection	Soybean, flaxseed, rice bran and cottonseed hydrolysates; sunflower peptide PADVTPEEKPEV; rice bran peptide AFDEGPWPK	Chemical antioxidant assays, cell-based oxidative stress models, selected animal studies; molecular docking available for some peptides	In vitro → Cell → Selected animal studies	[19,23,24,25,26,27,33,34,35,36,37,38,39,40,41]
Antihypertensive activity	ACE inhibition; bradykinin preservation; possible modulation of endothelial NO production	Hydrophobic or aromatic residues at the C-terminus, particularly Pro, Phe, Tyr and Trp; structural compatibility with ACE S1, S2 and S1′ subsites	Rice bran peptide YSK; ginger peptide VTYM; peptide-rich hydrolysates obtained from legumes and cereals	Molecular docking (selected peptides), ACE-inhibition assays, and animal studies evaluating blood-pressure reduction	In vitro → Animal studies; limited human validation	[40,42,43,44,45,46,47]
Antidiabetic potential	Inhibition of α-amylase, α-glucosidase and DPP-IV; modulation of GLUT2/SGLT1; activation of IR/IRS-1/AKT and GLUT4 signaling	Short sequences enriched in aromatic and/or charged amino acids facilitating enzyme interaction	Spent coffee ground peptides YGF and GMCC; hemp seed peptides; pseudocereal-derived inhibitory fractions	Molecular docking, enzymatic inhibition assays, cell culture studies, animal models	In silico → In vitro → Cell → Animal; limited clinical evidence	[18,49,50,51,52,53,54,55,65]
Immunomodulatory activity	Cytokine modulation; dendritic-cell maturation; predicted MHC-II interaction; regulation of innate and adaptive immune responses	Short motifs including YG, YGG, GLF, TPRK and related immunopeptide sequences	Rice-derived GBP1; database-curated cereal and legume peptide motifs	Bioinformatic prediction, database annotation, selected cellular validation studies; no direct MHC-II binding studies	In silico → Cell studies	[56,57,58]
Anticancer and chemopreventive effects	Apoptosis induction; autophagy; cell-cycle arrest; suppression of oncogenic transformation; modulation of histone acetylation	RGD motif and acidic C-terminal domain characteristic of lunasin; low-molecular-weight multifunctional sequences	Soybean lunasin; rice bran peptide EQRPR; walnut peptide CTLEW; black bean peptide fractions	Cell culture studies, mechanistic investigations, selected animal chemoprevention studies	Cell → Animal studies; no robust clinical evidence	[59,60,61,62,63,64,66,67]

Abbreviations: ACE, angiotensin-converting enzyme; DPP-IV, dipeptidyl peptidase-IV; NO, nitric oxide; ROS, reactive oxygen species. References are representative and correspond to sources already listed in the References section.

**Table 2:** Plant sources and agro-industrial by-products used for obtaining bioactive peptides, representative peptide sequences, production strategies and levels of experimental validation.

Plant Source or by-Product	Main Protein Precursor/Fraction	Representative Peptide(s) or Peptide Fraction	Identification Status	Production Approach	Reported Activity	Experimental Model/Validation Level	Key Considerations	References
Soybean and other major legumes	2S albumin, glycinin, β-conglycinin and related storage proteins	Lunasin, Vglycin, peptide-rich hydrolysate fractions	Sequence-confirmed peptides and peptide-rich hydrolysates	Enzymatic hydrolysis, gastrointestinal digestion, microbial fermentation	Antioxidant, anti-inflammatory, anticancer and antidiabetic effects	Cell studies, animal studies; limited human evidence	High research maturity; allergenicity assessment and clinical validation remain important	[60,66,68,74,92,93]
Chickpea, pea and lupin	Albumins and globulins	ADLPGLK; low-MW hydrolysate fractions; hypocholesterolemic lupin peptides	Sequence-confirmed peptides and peptide fractions	Enzymatic hydrolysis, simulated gastrointestinal digestion	Antioxidant, antiproliferative, lipid-regulatory and Nrf2-related effects	In vitro, selected animal studies	Sequence confirmation and in vivo validation remain incomplete in many reports	[23,69,70,71,72]
Rice and rice bran	Bran proteins and endosperm storage proteins	YSK, AFDEGPWPK, EQRPR, peptide-rich fractions	Sequence-confirmed peptides and hydrolysates	Enzymatic hydrolysis, fractionation, gastrointestinal digestion	ACE inhibition, antioxidant activity, antiproliferative effects	In vitro, cell studies	Promising preclinical evidence; translational validation still required	[40,63,75]
Maize, wheat and oat matrices	Zein, gluten/germ proteins, oat proteins	QLLPF, KELPPSDADW, oat-derived peptide fractions	Sequence-confirmed peptides and fractions	Hydrolysis, digestion and fractionation	Hepatoprotective, antiproliferative and anti-inflammatory effects; exercise-related outcomes reported for oats	In vitro, animal studies, selected human intervention studies (oats)	Gluten-related safety considerations apply to wheat-derived peptides	[64,77,78,94]
Quinoa and amaranth	Storage proteins of pseudocereals	DPP-IV inhibitory and α-glucosidase inhibitory peptide fractions	Fractionated peptide mixtures	Simulated digestion and enzymatic hydrolysis	Potential regulation of postprandial glycemia; chemopreventive activity	In vitro and cell studies	Clinical validation remains scarce despite promising biological effects	[65,79,80,81]
Oilseeds and nuts	Flaxseed, sunflower, perilla, chia, moringa and walnut proteins	PFFWLHHT, PADVTPEEKPEV, ISPRILSYNLR, CTLEW and related fractions	Sequence-confirmed peptides and peptide-rich fractions	Enzymatic hydrolysis, ultrasound-assisted processing, digestion	Antioxidant, anti-inflammatory, antihypertensive, neuroprotective, osteogenic and anticancer activities	In vitro, cell and selected animal studies	Sensory challenges (bitterness) and allergenicity should be considered	[19,23,84,85,86,87]
Potato peels	Patatin and protease inhibitors	Patatin-derived peptides and peptide-rich hydrolysates	Mainly hydrolysate fractions	Protein recovery followed by hydrolysis or extraction-assisted hydrolysis	Anti-inflammatory, gastroprotective and cardioprotective effects	In vitro and animal studies	Glycoalkaloids, compositional variability and safety monitoring remain critical	[17,89,95,96]
Spent coffee grounds	Residual proteins in SCG biomass	YGF, GMCC	Sequence-confirmed peptides	Fermentation, enzymatic hydrolysis, LC-MS/MS identification	α-Glucosidase inhibition and antidiabetic potential	Molecular docking, enzyme assays, cell studies	Standardization and biological validation remain important	[18,47,53]
Broccoli stems and tomato residues	Residual proteins from vegetable processing by-products	KSVLLKF (broccoli) and antioxidant peptide-rich fractions (tomato residues)	Sequence-confirmed peptides and hydrolysates	Enzymatic hydrolysis and fractionation	Wound-healing support, antioxidant activity	Cell studies	Underexplored source requiring additional validation	[26,91]
Algae and underutilized biomass	Algal proteins	ACE-inhibitory and radical-scavenging peptide fractions	Fractionated hydrolysates	Enzymatic hydrolysis and fractionation	Antioxidant and antihypertensive effects	In vitro and selected animal studies	Batch-to-batch variability and contamination control remain important	[29,97]

The table presents representative examples rather than an exhaustive inventory of all plant-derived bioactive peptides reported in the literature. Sequence-confirmed peptides are distinguished from peptide-rich fractions and hydrolysates to facilitate comparison of biological activities and levels of experimental validation. Reported activities are linked to the corresponding peptide or peptide preparation and reflect the highest level of evidence available in the cited literature, ranging from computational predictions and biochemical assays to cellular, animal and limited human studies.

**Table 3:** Technologies used during the production, recovery, purification, identification and biological validation of plant-derived bioactive peptides.

Process Stage	Technology	Principle	Main Advantages	Main Limitations	Suitable Matrices	Typical Outcome	Representative References
Peptide generation	Enzymatic hydrolysis	Food-grade proteases cleave parent proteins at enzyme-specific sites and release encrypted peptide sequences	High specificity, mild processing conditions, industrial familiarity and no toxic chemical residues	Enzyme cost, bitterness, dependence on hydrolysis degree and batch-to-batch variability	Legumes, cereals, seeds, nuts and plant by-products	Production of sequence-defined peptides and peptide-rich hydrolysates with antioxidant, antihypertensive and antidiabetic activities	[2,4,100,101,102]
Peptide generation	Sequential enzymatic hydrolysis	Two or more proteases are applied sequentially or simultaneously to broaden cleavage patterns	Greater peptide diversity and enhanced multifunctionality	More difficult process control and standardization	Legumes, cereals, oilseed meals and mixed protein matrices	Enhanced release of multifunctional peptides and low-MW fractions	[85,103]
Peptide generation	Microbial fermentation	Bacteria or fungi release peptides through endogenous proteolytic systems	Low cost, reduction in antinutritional factors, potential debittering and flavour improvement	Strong strain dependence, reproducibility and safety issues	Legumes, cereals, fermented beverages and spent coffee grounds	Simultaneous peptide generation and modification of sensory properties	[2,92,93,104,105,110,111]
Protein extraction and process assistance	Ultrasound-assisted extraction (UAE)	Acoustic cavitation disrupts plant structures and partially unfolds proteins, increasing enzyme accessibility	Shorter extraction/hydrolysis time and improved yield of low-MW fractions	Scale-up, energy input and process optimization remain challenging	Soybean meal, flaxseed and oilseed by-products	Enhanced protein recovery and improved susceptibility to hydrolysis	[7,35,84,106]
Protein extraction and process assistance	High hydrostatic pressure (HHP)	High pressure induces protein unfolding and structural relaxation before or during hydrolysis	Facilitates subsequent enzymatic release of peptides from compact protein structures	Specialized equipment and high cost	Potato, sweet potato and dense plant matrices	Improved enzymatic accessibility and peptide release	[29,107]
Peptide generation and extraction	Subcritical water hydrolysis	Pressurized hot water promotes hydrolysis without organic solvents or strong chemicals	Solvent-free processing approach with potential resource-efficiency advantages	Excessive temperature may degrade peptides and amino acids	Chia expeller and agro-industrial by-products	Recovery of peptide-rich fractions with antioxidant and ACE-inhibitory potential	[86,108]
Fractionation and purification	Membrane ultrafiltration	Separation according to molecular weight cut-off	Scalable and suitable for industrial processing	Membrane fouling and product losses	All hydrolysate matrices	Enrichment of low-MW bioactive fractions (<3–10 kDa)	[100,101,102,108]
Fractionation and purification	Chromatographic purification (SEC, RP-HPLC, IEC)	Separation based on size, hydrophobicity or charge	High purity and improved structure–activity interpretation	Cost and limited industrial scalability	Purified peptide fractions and sequence-confirmed peptides	Isolation of purified peptide sequences	[40,85,103,108]
Identification and characterization	LC-MS/MS	Peptide sequencing and identification	High sensitivity and structural resolution	Specialized equipment required	Purified peptides and peptide-rich fractions	Sequence confirmation by database searching and/or de novo sequencing	[18,26,53,56,57,58,65]
Biological validation	In vitro enzyme assays	Assessment of enzyme inhibition and antioxidant properties	Rapid screening and mechanistic insight	Limited physiological relevance	Purified peptides and hydrolysates	ACE, α-glucosidase, α-amylase and DPP-IV inhibition	[40,49,50,51,52,53,54,65]
Biological validation	Cell-based models	Evaluation of biological effects in cultured cells	Mechanistic and functional information	Limited systemic relevance	Purified peptides and fractions	Antioxidant, anticancer and immunomodulatory evaluation	[26,34,35,36,37,38,59,60,61,62,63]
Biological validation	Animal studies	Assessment of efficacy in vivo	Increased physiological relevance	Ethical and translational limitations	Selected peptide candidates	Antihypertensive, antidiabetic, neuroprotective and chemopreventive validation	[29,42,64,87,94]
Biological validation	Human intervention studies	Assessment of translational efficacy	Highest level of translational evidence	Limited availability for plant-derived peptides	Mainly protein preparations and selected oat-derived products	Evaluation of clinically relevant outcomes	[77,78]

The technologies listed above represent different stages of the peptide-production workflow and should not be considered functionally equivalent. Enzymatic hydrolysis, fermentation and subcritical water processing are primarily peptide-generating technologies, whereas ultrasound-assisted extraction and high hydrostatic pressure mainly act as pretreatment or process-enhancement approaches. Downstream processing typically involves fractionation, purification, analytical identification and subsequent biological validation.

**Table 4:** Translational barriers, mitigation strategies, evidence level and knowledge gaps for plant-derived bioactive peptides.

Challenge	Why It Matters	Examples/Affected Sources	Predominant Evidence Level	Possible Mitigation Strategy	Remaining Knowledge Gap	References
Bitterness and sensory defects	Reduces consumer acceptance and limits incorporation into beverages, dairy alternatives, bakery products and functional foods	Hydrolysates enriched in hydrophobic peptides; flaxseed, sunflower and oilseed protein hydrolysates	Mainly sensory studies and hydrolysis-process observations	Exopeptidase treatment, controlled fermentation, encapsulation, flavour masking, peptide fractionation	Limited sensory validation in complex food matrices and during long-term storage	[1,110,112,147]
Low gastrointestinal stability	Peptides may be degraded before reaching their target site or being absorbed	Antioxidant, ACE-inhibitory and DPP-IV inhibitory peptides	Simulated digestion (INFOGEST), biochemical stability studies	Gastrointestinal screening, encapsulation, cyclization, protective protein/polysaccharide matrices	Lack of standardized comparison among digestion studies and limited validation in vivo	[21,33,114,115,116]
Limited intestinal permeability	Stable peptides may still fail to cross the intestinal epithelium	Short peptides, peptide fractions and hydrolysates with demonstrated in vitro activity	Caco-2 and epithelial transport models; limited animal data	Permeability screening, carrier systems, peptide engineering	Scarcity of comparative transport studies and mechanistic absorption data	[21,114,115,116,117,148]
Poor systemic bioavailability	Biological activity observed in vitro may not translate into systemic efficacy in vivo	ACE-inhibitory, antioxidant and antidiabetic peptides	Selected animal studies; very limited pharmacokinetic studies in humans	Nanoencapsulation, nanoliposomes, chitosan nanoparticles, hydrogels, controlled-release systems	Lack of pharmacokinetic profiles, plasma stability studies and human bioavailability data	[21,33,66,148]
Allergenicity and food intolerance	Residual epitopes may induce IgE-mediated reactions or celiac responses in susceptible individuals	Soybean, peanut, lupin and wheat-derived proteins and peptides; hydrolysis-resistant gliadin fragments	Sequence analysis, allergen databases, selected experimental studies	Sequence screening, controlled hydrolysis, fermentation, thermal processing, use of low-allergen cultivars	Source-specific clinical validation and post-processing allergenicity assessment remain insufficient	[31,109,119,120,121,122,123,124,125,141,142,143,144,145,146,149]
Toxic plant constituents and contaminants	By-product streams may co-extract toxic compounds or processing contaminants	Potato glycoalkaloids, lectins, ribosome-inactivating proteins, canatoxin-like proteins	Toxicological studies and food-safety assessments	Fractionation, purification, raw-material quality control, contamination monitoring	Regulatory thresholds for peptide-rich ingredients obtained from by-products remain poorly defined	[17,96,127,128,129,130,131,132,133,134,135,136]
Insufficient clinical evidence	Limits the substantiation of health claims and commercial translation	Most antioxidant, antidiabetic, antihypertensive and anticancer peptides	Predominantly computational, biochemical, cellular and animal studies; only a limited number of controlled human intervention studies.	Randomized clinical trials, dose–response studies, long-term safety assessment	Effective doses, target populations and clinically relevant outcomes remain inadequately established	[17,21,67]
Process variability and lack of standardization	Different extraction and hydrolysis conditions generate different peptide profiles and bioactivities	Variability among cultivars, protein sources, enzymes and hydrolysis protocols	Comparative process studies and peptidomic analyses	Standardized production processes, peptide fingerprinting, validated bioassays, GMP-based manufacturing	Lack of harmonized protocols limiting comparison and regulatory acceptance	[116,150,151]
Challenges in peptide identification and structure–activity validation	Bioactivity is often attributed to fractions rather than sequence-confirmed peptides	Complex hydrolysates and peptide-rich fractions	LC-MS/MS identification, de novo sequencing, and bioinformatic prediction studies	Purification, de novo sequencing, synthetic peptide confirmation	Limited validation of predicted bioactivities using synthesized peptides	[56,57,58,131,132,133,134]
Sustainability and industrial scalability	Environmental benefits and commercial feasibility cannot be assumed solely from by-product utilization	Oilseed meals, potato peels, spent coffee grounds and other agro-industrial residues	Pilot-scale studies and process assessments	Process optimization, resource-efficiency analysis, circular-biorefinery approaches	Lack of techno-economic and life-cycle assessment data for most peptide production systems	[86,108,150,151]

Abbreviations: GMP, Good Manufacturing Practice; PDBAPs, plant-derived bioactive peptides. Evidence levels range from simulated digestion and biochemical assays to cellular, animal and limited human studies. The cited references are representative examples supporting each barrier, mitigation strategy and knowledge gap. Challenges related to bioaccessibility, intestinal permeability and systemic bioavailability are presented separately because they represent distinct stages influencing the biological efficacy of plant-derived bioactive peptides.

**Table 5:** Controlled human intervention studies involving plant-derived peptide preparations.

Intervention and Analytical Form	Design and Participants	Dose, Duration, and Comparator	Main Endpoint(s)	Main Finding	Interpretation, Limitations, and Reference
Pea protein hydrolysate; thermolysin digest enriched in <3 kDa peptides	Randomized, double-blind, placebo-controlled crossover pilot; 7 adults with hypertension	1.5 or 3 g/day for 3 weeks; placebo control	Systolic blood pressure (SBP)	The 3 g/day dose reduced SBP versus placebo by 5 and 6 mmHg in weeks 2 and 3, respectively; the lower dose was not reported as effective.	Very small, short pilot; complex peptide fraction; no attribution to a specific sequence. V5; [157]
Black soy peptide supplement; complex peptide mixture	Randomized, double-blind, placebo-controlled parallel trial; 100 participants with prehypertension or stage I hypertension	4.5 g/day for 8 weeks; placebo	SBP, diastolic blood pressure (DBP), and oxidative-stress markers	The adjusted SBP decrease was greater with the supplement than with placebo (−9.69 ± 12.37 vs. −2.91 ± 13.29 mmHg; p = 0.015); selected oxidative-stress markers also changed.	Short intervention; multicomponent preparation; the effect cannot be assigned to an individual peptide sequence. V5; [158]
Black soy peptide supplement; complex peptide mixture	Double-blind, randomized, placebo-controlled trial; 80 overweight or obese adults randomized	4.5 g/day for 12 weeks; placebo	Body weight, body mass index (BMI), and body-fat measures	The intervention group showed greater reductions in body weight, BMI, and body-fat measures than the placebo group after 12 weeks.	Attrition and short follow-up; peptide composition was complex; no sequence-specific causal attribution. V5; [159]
Lunasin-enriched soy extract; not purified lunasin	Triple-blind, placebo-controlled crossover trial; 31 adults (mean age approximately 61 years)	335 mg/day for 8 weeks; placebo; 3-week washout	Serum lipids, glucose, insulin resistance, blood pressure, BMI, and waist circumference	No statistically significant improvements in the assessed cardiometabolic risk factors were observed.	Small trial; the intervention was an enriched extract rather than purified lunasin; null result limits clinical claims. V5; [160]
Vicia faba-derived peptide network (NPN_1); defined commercial peptide-rich preparation	Randomized parallel trial; 30 healthy young men undergoing 7 days of single-leg immobilization and 14 days of remobilization	10 g twice daily for 21 days; isonitrogenous milk-protein concentrate	Quadriceps size and myofibrillar protein-synthesis rates	Changes in muscle size did not differ between groups; myofibrillar protein synthesis was higher with NPN_1 during remobilization.	Active rather than placebo comparator; healthy young men only; proprietary mixture prevents attribution to a single sequence. V5; [161]
Hemp seed protein plus hydrolysate-derived peptide fraction (HSP+); compared with intact hemp protein	Randomized, double-blind crossover trial; 35 adults with mild hypertension	Three 6-week periods: 50 g casein/day, 50 g hemp protein/day, or 45 g hemp protein + 5 g peptide fraction/day; 2-week washouts	24-h ambulatory SBP and DBP; ACE, renin, and nitric oxide biomarkers	Both hemp-protein interventions lowered 24-h BP versus casein, with the largest reductions after HSP+; biomarker differences did not consistently distinguish HSP+ from intact hemp protein.	High protein doses; intact hemp protein was also active, so the independent contribution of the 5 g peptide fraction is uncertain. V5; [162]

The table includes controlled human interventions in which a plant-derived peptide mixture, hydrolysate, peptide-enriched extract, or peptide network was administered. None of the trials establishes clinical efficacy of an individually purified sequence; even the lunasin trial evaluated an enriched soy extract rather than purified lunasin. ACE, angiotensin-converting enzyme; BMI, body mass index; DBP, diastolic blood pressure; HSP, hemp seed protein; HSP+, hemp seed protein supplemented with hydrolysate-derived peptides; SBP, systolic blood pressure; V5, controlled human intervention evidence.

## Data Availability

No new data were created or analyzed in this study. Data sharing is not applicable to this article.

## Supplementary Materials

The following supporting information can be downloaded at: https://www.mdpi.com/article/10.3390/molecules31162866/s1; Table S1. Database-specific literature search strategy used for the structured narrative review. Table S2. Study-level analytical and experimental characteristics of representative plant-derived bioactive peptide preparations discussed in this review.
