# Supplementary material for: Comprehensive Insights into Plant-Derived Bioactive Peptides: Sources, Technological Strategies, and Health Implications
Source: Molecules. 2026 Aug 17;31(16):2866. doi: 10.3390/molecules31162866 (PMC13515153; doi:10.3390/molecules31162866)
Supplement: Supplementary file 1 [file molecules-31-02866-s001.zip › molecules-4448745-supplementary.pdf]

Supplementary Table S1. Database-specific literature search strategy used for the structured narrative review.

Database	Search field(s)	Search string used	Filters applied
**Scopus**	Title, Abstract, Keywords	TITLE-ABS-KEY (("plant-derived bioactive peptide*" OR "plant bioactive peptide*" OR "plant protein hydrolysate*") AND ("enzymatic hydrolysis" OR fermentation OR digestion) AND (antioxidant OR antihypertensive OR antidiabetic OR immunomodulatory OR anticancer OR bioavailability OR toxicity OR "functional food*"))	English language; Articles and Reviews; 2015–2026*
**Web of Science Core Collection**	Topic (TS)	TS=(("plant-derived bioactive peptide*" OR "plant protein hydrolysate*") AND ("enzymatic hydrolysis" OR fermentation OR digestion) AND (antioxidant OR antihypertensive OR antidiabetic OR immunomodulatory OR anticancer OR bioavailability OR toxicity))	English language; Articles and Reviews; 2015–2026*
**PubMed**	Title/Abstract	(("plant-derived bioactive peptides"[Title/Abstract] OR "plant protein hydrolysates"[Title/Abstract]) AND ("enzymatic hydrolysis"[Title/Abstract] OR fermentation[Title/Abstract] OR digestion[Title/Abstract]) AND (antioxidant OR antihypertensive OR antidiabetic OR immunomodulatory OR anticancer OR bioavailability))	English language; 2015–2026*
**ScienceDirect**	Title, Abstract, Keywords	"plant-derived bioactive peptides" AND ("enzymatic hydrolysis" OR fermentation OR digestion) AND (antioxidant OR antihypertensive OR antidiabetic OR immunomodulatory OR anticancer)	Research articles and reviews; English language
**Google Scholar**	Full-text search	"plant-derived bioactive peptides" "enzymatic hydrolysis" antioxidant antihypertensive antidiabetic functional foods	Results sorted by relevance; first 200 records screened for each predefined query

*Older landmark publications were additionally included when they provided original mechanistic, analytical or safety-related information.

Older landmark publications published before 2015 were additionally included when they provided original mechanistic, analytical, methodological, or safety-related information directly relevant to the scope of this review. Search strings were adapted to the syntax and indexing system of each database while preserving the same conceptual search strategy.

Supplementary Table S2. Study-level analytical and experimental characteristics of representative plant-derived bioactive peptide preparations discussed in this review.

Plant source	Peptide / preparation	Analytical status	Peptide preparation	Experimental model	Bioactivity endpoint	Major analytical limitations	Main reference
Soybean	Lunasin	Sequence-confirmed peptide	Purified peptide	Cell, animal, limited human	Anticancer, anti-inflammatory	Human studies evaluated enriched extracts rather than purified lunasin	[59–62,160]
Rice bran	YSK	Sequence-confirmed peptide	Purified peptide	Enzyme assay	ACE inhibition	No direct human validation	[40]
Rice bran	AFDEGPWPK	Sequence-confirmed peptide	Purified peptide	Antioxidant assays	Antioxidant activity	Mainly in vitro evidence	[41]
Rice bran	EQRPR	Sequence-confirmed peptide	Purified peptide	Cell culture	Antiproliferative activity	Cell models only	[63]
Ginger	VTYM	Sequence-confirmed peptide	Purified peptide	Docking + ACE assay	ACE inhibition	Docking does not establish direct binding	[40]
Potato peel	Patatin-derived hydrolysate	Peptide-rich hydrolysate	Hydrolysate	Cell / animal	Anti-inflammatory, cardioprotective	Individual active sequences not identified	[17,89,95]
Spent coffee grounds	YGF	Sequence-confirmed peptide	Fermentation-derived peptide	Docking + enzyme assay	α-Glucosidase inhibition	No animal or clinical validation	[18,53]
Spent coffee grounds	GMCC	Sequence-confirmed peptide	Fermentation-derived peptide	Docking + enzyme assay	α-Glucosidase inhibition	No animal or clinical validation	[18,65]
Broccoli stems	KSVLLKF	Sequence-confirmed peptide	Purified peptide	Cell culture	Wound healing	No animal confirmation	[97]
Walnut	CTLEW	Sequence-confirmed peptide	Purified peptide	Cell culture	Anticancer activity	Cytotoxicity only; no clinical evidence	[64]
Flaxseed	Peptide-rich fraction	Peptide fraction	Hydrolysate fraction	Antioxidant assays	Antioxidant activity	Composition incompletely characterized	[33,84]
Hemp seed	Peptide-rich hydrolysate	Peptide fraction	Hydrolysate	Enzyme assay / animal	ACE inhibition	Fraction rather than	[50,51]

						defined peptide	
Rice bran	EQRPR	Sequence-confirmed peptide	Purified peptide	Cell culture	Antiproliferative activity	Cell models only	[63]

Analytical status distinguishes sequence-confirmed peptides from peptide-rich fractions and crude hydrolysates. Reported biological activities correspond to the experimental model used in the cited study and should not be interpreted as equivalent levels of evidence. Computational predictions, biochemical assays, cell-based studies, animal experiments, and human intervention studies provide different levels of validation and should be interpreted accordingly.
